# Novel function for the p38-MK2 signaling pathway in circulating CD1c+ (BDCA-1+) myeloid dendritic cells from healthy donors and advanced cancer patients; inhibition of p38 enhances IL-12 whilst suppressing IL-10

**DOI:** 10.1002/ijc.28398

**Published:** 2013-11-20

**Authors:** Hester A Franks, Qunwei Wang, Stephanie J Lax, Mary K Collins, David Escors, Poulam M Patel, Andrew M Jackson

**Affiliations:** 1 Host:Tumour Interactions Group, Academic Unit of Clinical Oncology University of NottinghamUnited Kingdom; 2 MRC Centre for Medical Molecular Virology, University College LondonUnited Kingdom; 3 Immunomodulation group, Navarrabiomed-Fundacion Miguel Servet National Health Service of NavarrePamplona, Navarra, Spain

**Keywords:** dendritic cell, signaling, IL-12, Th1, p38

## Abstract

There is growing interest in myeloid (my) dendritic cells (DC) as an alternative to monocyte-derived DC (moDC) for immunotherapy. However, in contrast to moDC, little is known regarding the effect of malignancy on the function, abundance or use of intracellular signaling pathways in myDC. Understanding the molecular detail of circulating myDC is therefore important for future use in advanced cancer. Advanced cancer patients had similar numbers of circulating myDC to cancer-free patients and healthy individuals, and secreted similar levels of IL-1β, IL-6, IL-10, IL-12 and IL-23. However, myDC from some patients failed to secrete the Th1-cytokine IL-12. Surprisingly, inhibiting p38 (p38i) signaling (using BIRB0796 or SB203580) markedly increased IL-12 secretion by myDC. This is in complete contrast to what is established for moDC where inhibiting p38 ablates IL-12. Interestingly, this was specific to IL-12, since IL-10 was suppressed by p38i in both DC types. The opposing effect of p38i on IL-12 was evident at the transcriptional level and in both DC types was mediated through the p38-MK2 pathway but did not involve differential phosphorylation of the distal Rsk kinase. Importantly, where patient myDC did not secrete IL-12 (or after treatment with suppressive melanoma lysate), p38i restored IL-12 to normal levels. In contrast to p38, inhibiting the other MAPK pathways had similar consequences in both DC types. We show for the first time the differential use of a major intracellular signaling pathway by myDC. Importantly, there are sufficient circulating myDC in advanced cancer patients to consider development of adoptive immunotherapy.

**What’s new?:**

Dendritic cells (DCs) govern antigen specificity in T cells. DCs also secrete cytokines that regulate T-cell responses. This study explores the potential of circulating myeloid dendritic cells (myDC) for cancer immunotherapy. The authors examined intracellular signalling and cytokine secretion in myDCs, and found that when p38 MAPK is inhibited in these cells, IL-12p70 production is enhanced and IL-10 is suppressed. In contrast, monocyte-derived DCs (moDCs) require p38 MAPK for IL-12p70 production. These differences in intracellular signalling indicate that immunotherapy with myDCs may induce more potent anti-tumour immunity in combination with MAPK inhibitors.

Dendritic cells (DC) govern T-cell antigen-specificity, activation and polarization and their secretion of Interleukin (IL)-12p70 (IL-12) induces Th1 and cytotoxic T cell (CTL) responses.^1^ Their role in anti-tumor immunity led to investigation of monocyte-derived DC (moDC) for immunotherapy. Circulating CD1c+ myeloid DC (myDC) are a physiological population of human DC which do not require *in vitro* generation and therefore have technical advantages over moDC.^2^,^3^ In addition, the potential immunotherapeutic benefits of myDC include more potent induction of T cell responses^4^ and more efficient chemotaxis toward T-cell chemokines.^5^–^7^ Whilst CD1c+ myDC share many general characteristics with moDC including cross-presentation, response to danger and priming T-cells,^4^,^8^,^9^ it has become increasingly clear that they do not function in exactly the same way and need to be studied in their own right. Key considerations yet to be addressed are whether there are sufficient myDC to use for immunotherapy in advanced cancer patients and whether they have normal function when isolated from the blood of cancer patients.

In order to design a clinically effective DC therapy, the ability to enhance Th1 polarization by increasing IL-12 secretion and suppress Treg induction *via* a reduction in IL-10 would be advantageous. We have studied other intracellular signaling pathways and demonstrated a novel role for the ATM DNA repair pathway in regulation of IL-23 and Th17 polarization in myDC and moDC.^10^ The MAPK pathways are critically involved in DC cytokine secretion and their role in determining the pattern of cytokine release after activation has been extensively studied in moDC.^11^–^18^ In contrast to moDC, MAPK signaling in human circulating myDC has not yet been studied, and whether intracellular signaling is the same in moDC and myDC is unknown. If these pathways are to be targeted to enhance Th1/suppress Treg polarization in the setting of a DC vaccine, it is crucial to have a full understanding of how they function in the particular DC subset being used.

In addition to their role for cytokine production in DC, the MAPK pathways are of substantial current clinical interest for direct targeting in disease. Small-molecule p38 inhibitors are in clinical trials in cancer^19^, rheumatoid arthritis^20^, chronic obstructive pulmonary disease^21^ and neuropathic pain,^22^ although the results in autoimmune disease in particular have been disappointing. Lentiviral targeting of MAPK pathways in DC is being investigated for the treatment of cancer^23^ and autoimmune diseases.^24^ These studies derive from observations of abnormalities in MAPK pathways in a range of diseases and pre-clinical studies.^25^–^28^ Targeting the RAS-RAF-MEK Extracellular signal-regulated kinase (ERK) pathway with small molecule inhibitors is clinically beneficial in BRAF-mutated melanoma.^29^,^30^ With the explosion of interest in molecular targeting, it is important to understand the potential effects of these therapeutic strategies outside the intended target tissue.

This study identifies for the first time key differences in function of the MAPK pathways in myDC compared with moDC. Whilst the MEK/ERK pathway served similar roles for cytokine production, marked differences were observed between myDC and moDC for p38 MAPK. In particular, the p38 pathway served a negative regulatory role for IL-12 production in myDC in contrast to the canonical positive role in moDC.^11^–^13^ Interestingly, this was IL-12-specific, as p38 inhibition (p38i) reduced IL-10 (accepted to inhibit anti-cancer T cell responses) in both types of DC. In Stage 4 cancer patients, we established that there are sufficient numbers of circulating myDC for therapeutic vaccine use. Importantly, we demonstrated that even in myDC from advanced cancer patients that are severely impaired in their ability to produce IL-12, p38i restores secretion to normal levels whilst suppressing IL-10. Appreciating the differential use of the major intracellular signaling pathways in myDC has clear implications for the clinical application of MAPK inhibitors and for the design of immunotherapies in oncology.

## Material and Methods

### Reagents

Endotoxin-free reagents were used throughout. All media were based on RPMI 1640 with l-glutamine (Sigma Gillingham, UK). DC medium contained vol/vol 10% fetal calf serum (FCS) and 1% sodium pyruvate (Sigma), T cell medium vol/vol 10% FCS, 1% *4-(2-hydroxyethyl)-1-piperazineethanesulfonic acid* (HEPES), 1% sodium pyruvate, 1% nonessential amino acids and 20 µM 2-mercaptoethanol (Sigma) and MM418 medium 10% vol/vol FCS (PAA Yeovil, UK). Cytokines were recombinant human (rh) IL-4 (1,000 U/ml, Immunotools Friesoythe, Germany), rh granulocyte-macropahge colony stimulating factor (GM-CSF) (1,000 U/ml, Peprotech London, UK), rhIL-2 (50 U/ml) and rhIL-10 (Miltenyi Bergisch Gladbach, Germany). Ultrapure toll-like receptor (TLR) agonists were R848 (TLR7/8, 2.5 µg/ml, Invivogen San Diego, California) and Poly I:C (TLR3, 20 µg/ml, Sigma). T-cells were stimulated with mouse anti-human CD3 (OKT3, 1 µg/ml, produced in house) and CD28 (5 µg/ml, BD Pharmingen Oxford, UK). Mouse anti-human CD1c Phycoerythrin (PE) (Miltenyi Biotech), CD14 Fluorescein isothiocyanate, CD4 PE (both BD), CD45RA FITC (eBioscience Hatfield, UK) and CD45RO PE (Biolegend London, UK) were used with matched isotype controls. Small molecule inhibitors of MAPK (MAPKi) (UO126 Cell Signaling Boston MA, SB203580 and SP600125 Calbiochem Darmstadt, Germany) were used at 10 µM, BIRB0796 (Selleckchem Newmarket, UK) was used at 0.1–1.0 µM and MK2 inhibitor III (Calbiochem) at 1–3 µM.

### Human participants

Heparinized blood was obtained from volunteers after written informed consent with ethical approval from Nottingham University Ethics Committee and Nottingham Research Ethics Committee 2. Melanoma and bowel cancer patients were recruited from Oncology Outpatient Clinics at Nottingham University Hospitals NHS Trust. Exclusion criteria were recent (<3 months) chemotherapy, any prior immunotherapy or anemia.

### MyDC and moDC

Peripheral blood mononuclear cells (PBMC) were separated from heparinized blood by centrifugation over Histopaque 1077 (Sigma). MyDC were isolated using Miltenyi CD1c myDC kit as per manufacturer instructions. Briefly, B cells were depleted (anti-CD19 microbeads) before positive selection of CD1c+ cells (routinely >95% pure by flow cytometry). MyDC were rested for 2 hr at 37°C before further treatment. CD1c− cells were incubated with anti-CD14 microbeads (Miltenyi), CD14+ monocytes (>95% pure) selected and resuspended at 0.5 × 10^6^/ml in DC medium, IL-4 and GM-CSF. Half-volume medium/cytokines were added on day 3 and immature moDC harvested at day 5–6.

### *In vitro* stimulation

Immature DC (10^4^/well) were plated in DC medium with GM CSF. GM-CSF was required for viability of myDC, and included with moDC for direct comparison. The presence of GM-CSF did not alter cytokine production by moDC, nor did it alter the effect of MAPKi in moDC (not shown). DC were treated with MM418 tumor-derived lysate (7.5 × 10^5^ cells/ml) for 1 hr, MAPKi for 1 hr then poly I:C/R848 for 24 hr. To investigate the role of DC-derived IL-10 in regulation of IL-12, rhIL-10 was added (0.1–1.0 ng/ml) 4 hr after TLR stimulation (based on published data regarding timing of DC secretion of IL-10). Supernatants were harvested and assayed by ELISA for IL-12p70 (BD biosciences), IL-23 (eBioscience), IL-1β, IL-10 (both R&D Abingdon, UK) and IL-6 (Immunotools).

### Preparation of tumor derived lysate

MM418 cells were seeded into a 10-layer factory flask (Corning Costar Amsterdam, The Netherlands), harvested by trypsinization after 3 days, washed and resuspended at 1 × 10^7^/ml in RPMI 1640. Lysate was prepared by five cycles of freeze-thaw as previously described.^18^ Mycoplasma testing was negative.

### Quantitative PCR

MyDC and moDC were plated in DC medium with GM-CSF (10^5^/well in 48-well plates) and stimulated with poly I:C/R848 ± pre-treatment with SB203580 for 1 hr. At 12 hr, cells were harvested into cold PBS, RNA isolated (Nucleospin RNAII kit, Macherey-Nagel Düren, Germany) and cDNA prepared (GoScript Reverse Transcription system, Promega Madison, WI). Taqman quantitative PCR was carried out for IL12A, IL12B and IL23A with TOP1 as housekeeping gene (Applied Biosystems Paisley, UK, TOP1 Hs00243257_m1, IL-12A/IL-12p35 Hs00168405_m1, IL-12B/IL-12p40 Hs00233688_m1, IL-23A/IL-23p19 Hs00372324_m1), with Mastermix (Primer Design Southampton, UK) on a Stratagene MX3005P and analyzed with Stratagene software. PCR conditions were 10 min/95°C then 50 cycles of 15 sec/95°C and 60 sec/60°C. Quantification was done by δδCT method where δCT = (gene of interest CT) − (TOP1 CT), δδCT calculated with poly I:C/R848 as reference condition and fold change in gene expression = 2^−δδCT^. Between 450–690 ng of RNA was obtained from 10^5^ DC per condition, with a 260/280 ratio of 1.7–2.0. Efficiency of amplification of all genes was >90%.

### Polarization of naive T cell responses

DC culture supernatants were generated from 10^5^ cells stimulated with poly I:C/R848 ± SB203580. At 12 hr, cells were washed to remove TLR agonists and p38i, and fresh medium added. Supernatants were harvested at 24 hr. Cell-free “mock” supernatants were generated to control for carry-over of TLR agonists/p38i. Naïve T cells (CD4+CD45RA+) were isolated using CD4+ T cell isolation kit II and CD45RA microbeads (Miltenyi) (purity> 95%). T cells (2 × 10^5^/well) were cultured with IL-2 in plates coated with anti-CD3 (1 µg/ml) with 25% DC culture supernatant and anti-CD28 (5 µg/ml). On day 5, cells were harvested and rested for 3 days. Cells were re-stimulated (2 × 10^5^/well) with anti-CD3/CD28/IL-2 and Golgi-Plug (BD Pharmingen). After 20 hr, cells were fixed (0.5% formaldehyde), permeabilized (Perm Buffer, Biolegend) and incubated with mouse anti-human Interferon (IFN)γ AF647 (BD Pharmingen), IL-17 FITC (eBioscience) and rat anti-human IL-10 PE (BD Pharmingen). CompBeads (Beckman Coulter High Wycombe, UK) were used for compensation and fluorescence-minus-one (FMO) controls with matched isotypes were used.

### Western blotting

MoDC (10^6^/well, 12-well plates) and myDC (10^5^/well, 96-well plates) were plated in DC medium containing GM-CSF. For experiments directly comparing moDC and myDC, cells were plated at 10^5^/well in 96-well plates. Cells were rested for 2 hr, treated with MAPK inhibitor for 1 hr, stimulated with Poly I:C/R848 then harvested, washed and lysed in RIPA buffer (Sigma) containing protease inhibitor cocktail, phosphatase inhibitor cocktail 2 and 3 and Benzonase endonuclease (all Sigma) on ice for 1 hr. Debris was removed by centrifugation and samples frozen at −20°C.

Proteins were resolved by electrophoresis in 10% Tris/glycine gels or 4–12% Tris/glycine gradient gels (Novex Paisley, UK) and transferred onto nitrocellulose. Membranes were blocked with 5% milk (wt/vol) in PBS-0.1% (vol/vol) Tween 20 for 1 hr, probed with primary antibodies overnight at 4°C and secondary antibodies for 1 hr at room temperature in 1% milk (wt/vol) in PBS-0.1% (vol/vol) Tween 20. Primary antibodies were rabbit anti-human phospho-MK2(T334) (#3007), phospho-p38(T180/T182) (#4511), phospho-ERK1/2(T202Y204) (#9101), phospho-Rsk(T359/S363) (#9344), phospho-Rsk(T573) (#9346), phospho-Rsk(S380) (#9335) and total Rsk(#9355) (Cell Signaling Technology) and mouse anti-human β-actin (Sigma). Binding of secondary antibodies (infrared-dye conjugated (Licor Lincoln, Nebraska): donkey anti-rabbit-800 and anti-mouse-680) was detected by Licor Odyssey. For semi-quantitative analysis, Odyssey software was used and intensity normalized to loading control.

### Whole blood DC enumeration assay and flow cytometry

300 µl heparinized whole blood was incubated with control or active cocktail of antibodies for 15 min at room temperature. Control cocktail contained mouse anti-human CD19 PECy5 (BD), CD14 PECy5 (Beckman Coulter), IgG1 FITC (Miltenyi) and IgG2a PE (Miltenyi), active cocktail contained CD19 PECy5, CD14 PECy5, CD303 FITC (Miltenyi) and CD1c PE (Miltenyi). 300 µl Cal-Lyse (Invitrogen) was added and incubated for 10 min, followed by 3 ml distilled water and incubation for 5 min. Cells were pelleted by centrifugation, resuspended in flow cytometry (FACS) buffer, flowcount fluospheres (Beckman Coulter) added for absolute enumeration and at least 10^6^ events acquired. Comp-beads (BD) were used to set compensation. Optimization and validation of protocol was carried out to ensure reproducibility prior to enumeration of patient DC.

### Statistics

Cytokine levels are presented as mean ± standard deviation and parametric tests were used where possible with transformations to achieve normality attempted before non-parametric tests used. For analysis of MAPKi on healthy donor cytokine levels, drug-treated cytokine levels are expressed as fold change compared to TLR agonist alone and myDC and moDC compared by one-way ANOVA with Dunnetts post-test correction. For patient data, cytokine analysis was done after square root transformation to achieve normality (confirmed by Komogorov–Smirnov test). Baseline cytokine was compared using one-way ANOVA with Bonferroni correction of *p* values. MAPKi and tumor derived lysate (TDL) effect was analyzed using two-tailed paired students’ *t*-test with Bonferroni correction. Combined TDL and SB203580 effect on IL-12 was assessed using repeated measures ANOVA with Bonferroni post-test. Circulating DC counts between groups was compared using unpaired two tailed students’ *t*-test with Welch’s correction for unequal variances. On all figures *p < 0.05, **p < 0.005.

## Results

### Advanced cancer patients have normal numbers of DC

**Figure 1:**
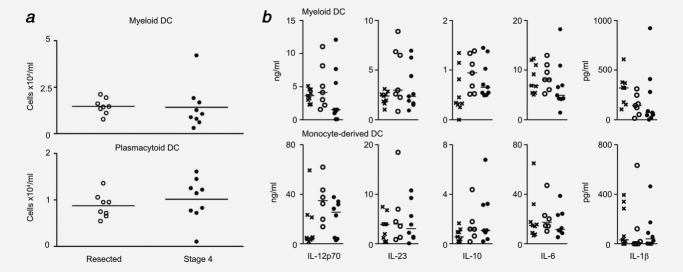
Number and baseline function of cancer patient DC. (*a*) MyDC (CD1c+) and plasmacytoid DC (pDC) (CD303+) were enumerated in heparinized whole blood from fully resected (*n* = 8, open circles) and Stage 4 (*n* = 9, filled circles) cancer patients using Countbeads to enable direct absolute quantification. Shown is cells × 10^4^/ml for each patient and bar at mean for the group. (*b*) MyDC and moDC from healthy donors (*n* = 9, crosses), fully resected (*n* = 7 myDC, *n* = 6 moDC, open circles) and advanced cancer patients (*n* = 9 myDC, *n* = 8 moDC, filled circles) were stimulated in triplicate with poly I:C/R848 for 24 hr and cytokine production analyzed by ELISA. Shown is mean cytokine production for each donor with bar at median for the group.

To determine whether circulating human myDC from cancer patients are a potential therapeutic option for immunotherapy, we established the baseline number and function of DC in patients with fully resected and advanced (Stage 4) cancer. Patient characteristics are shown in Table1. The staining protocol and analysis strategy were optimized on healthy donors (gating strategy Supporting Information Fig. 1A). Validation experiments and analysis demonstrated reproducible staining and gating (Supporting Information Figs. 1B and 1C). Circulating myDC and plasmacytoid DC numbers (Fig. 1*a*) in advanced cancer patients (*n* = 9) were the same as patients with fully resected cancer (*n* = 8) (*p* = 0.89 and *p* = 0.45, respectively) and healthy donors from optimization experiments.

**Table 1:** Patient demographic and disease-specific details

	Fully resected	Stage 4
Number	9	9
Age (years, median (range))	57 (35–65)	63 (55–85)
Sex (male:female)	7:2	5:4
**Stage of disease at original diagnosis**		
Stage 1	2	0
Stage 2	4	5
Stage 3	3	0
Stage 4	0	4
Time since primary resection (months, median (range))^1^	37 (1–75)	30 (0–82)
Time since metastatic disease diagnosed (months, median (range))	n/a	6 (1–18)
**Sites of metastatic disease present**^2^	n/a	
Subcutaneous		3
Lung		6
Liver		7
Lymph node		5
Other^3^		2
**Previous treatments**		
Dacarbazine	0	3
Capecitabine/5FU + oxaliplatin	0	2
Radiotherapy	0	1

1 Primary tumor not resected for two patients in Stage 4 group.

2 All Stage 4 patients had >1 site of metastatic disease.

3 Other: 1 patient with brain metastases and retroperitoneal metastases, 1 patient with spleen metastases.

### DC from advanced cancer patients secrete normal levels of cytokines

Recent work has shown that dual TLR activation of DC is far more effective at inducing cytokine secretion than single TLR ± IFNγ or cytokine cocktails.^8^,^31^ Combinations including a TLR7/8 agonist are key to this effect, and therefore poly I:C (TLR3) and R848 (TLR7/8) stimulation was used throughout. There was no significant difference between baseline cytokine production between healthy donors and fully resected or advanced cancer patients for any of the cytokines measured for myDC or moDC (Fig. 1*b*) except for IL-1β between healthy and resected patients for myDC. However, whilst as a group the cytokine production from Stage 4 patients’ myDC was normal (Fig. 1*b*, top), some patients exhibited markedly reduced cytokine levels, particularly for IL-12.

### Role of MAPK pathways for cytokine production by human DC

**Figure 2:**
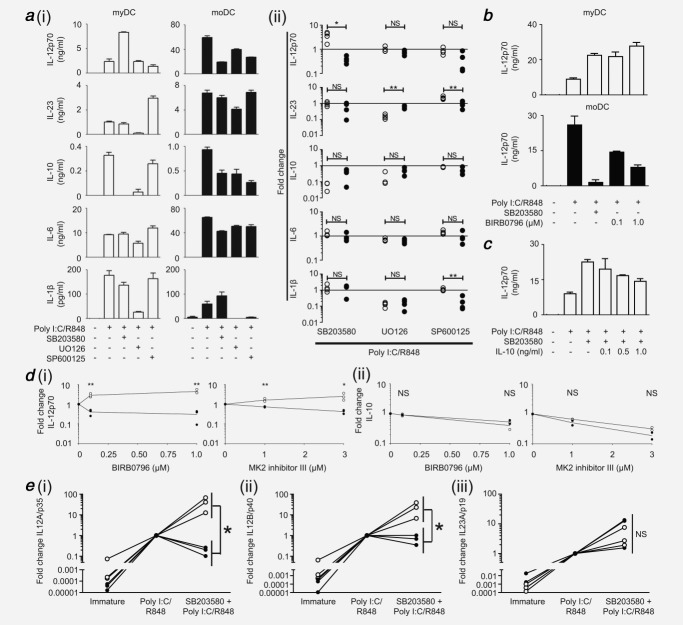
Role of MAPK pathways in DC cytokine secretion for healthy donors. (*a*) MyDC and moDC were stimulated with poly I:C/R848 1 hr after pre-treatment with inhibitors targeting p38 (SB203580), MEK (UO126) or JNK (SP600125). After 24 hr, cytokine production was determined by ELISA. (*i*) Absolute cytokine levels for a representative donor of four, open bars = myDC, filled bars = moDC. (*ii*) Summary of fold change in cytokine production for each drug compared to poly I:C/R848 alone for four independent donors, open circles = myDC, filled circles = moDC. (*b*) DC were treated and analyzed as in (*a*) but with the alternate p38 inhibitor BIRB0796 compared to SB203580 or no drug. Shown is one representative donor of three. (*c*) MyDC were treated as in (*a*) with addition of rhIL-10 at 4 hr after TLR stimulation. Shown is one representative donor of two. (*d*) MyDC (open circles) or moDC (filled circles) were treated with the indicated concentrations of the p38 inhibitor BIRB0796 or MK2 inhibitor III for 1 hr prior to TLR (poly I:C/R848) stimulation and analysis as in (*a*). Shown is fold change compared to no drug for three independent donors. Statistical significance is between myDC and moDC for each drug dose. (*e*) DC were stimulated with poly I:C/R848 with or without pre-treatment with p38 inhibitor SB203580 (10 µM) for 1 hr. Quantitative PCR was used to measure expression of IL-12A/p35 (*i*), IL-12B/p40 (*ii*) and IL-23A/p19 (*iii*) at 12 hr after TLR stimulation and standardized to the TOP-1 gene. Fold change is relative to poly I:C/R848 for each donor. Data are given for three independent donors comparing myDC (open circles) and moDC (filled circles). In all parts, bars are mean of experimental triplicate for a donor ± SD. * *p* < 0.05, ** *p* < 0.005, NS = non-significant.

There is a paucity of work concerning intracellular signaling pathways in myDC. Because of the increased clinical use of kinase-inhibitors and emerging immunotherapeutic interest in myDC, we investigated the function of MAPK pathways for cytokine production using healthy donors. In agreement with previous studies,^11^–^13^ the p38 pathway was crucial for IL-12 production by moDC with secretion substantially suppressed upon p38 inhibition (p38i) [Fig. 2*a*(*i*,*ii*)]. In marked contrast, the p38 pathway in myDC was a potent repressor of IL-12 as a pronounced increase in cytokine secretion was observed following p38i. Despite the common p40 subunit, p38 did not regulate IL-23 in either DC type [Fig. 2*a*(*i*,*ii*)]. IL-6 and IL-1β were similarly not regulated by p38; however, it positively regulated IL-10 production in both moDC and myDC.

In contrast to p38, myDC and moDC responded similarly to inhibition of the MEK-ERK pathway [Fig. 2*a*(*i*,*ii*)] although differences in the magnitude of effect were evident. MEK-ERK had no role in either DC type for IL-12 but was required for IL-1β, IL-23 and IL-10 production. In myDC, the c-Jun N-terminal kinase (JNK) pathway played no role for most cytokines studied. Inhibition with SP600125 suggested that JNK was repressive for IL-23. Conversely, in moDC JNK appeared to play a greater role, as SP600125 suppressed production of IL-12, IL-10 and IL-1β.

An alternate p38i, BIRB0796, was used to confirm that the effects observed were due to differential utilization of p38, and not off-target effects of SB203580. At 0.1 µM, BIRB0796 inhibits p38α and β without known off-target effects^32^ whilst at 1.0 µM it also inhibits p38γ and δ, with some effect on JNK2. In myDC, BIRB0796 (0.1µM) produced a similar increase in IL-12 production to that seen with SB203580 (Fig. 2*b*). In moDC, BIRB0796 (0.1 µM) halved IL-12 production, with further suppression at 1.0 µM (Fig. 2*b*). Whilst BIRB0796 did not suppress IL-12 from moDC as much as SB203580, the differential effect on IL-12 production between myDC and moDC was similar.

### Loss of IL-10 is not responsible for myDC IL-12 production following p38i

IL-10 can inhibit IL-12 production^33^ and we observed that p38-inhibited moDC retained some IL-10 secretion, whilst p38-inhibited myDC did not. We therefore confirmed that loss of IL-10 secretion was not responsible for enhanced IL-12 after p38i in myDC. IL-10 production by DC is first detected 4 hr after TLR stimulation, so IL-10 was added to p38-inhibited, activated myDC at this time point, at concentrations typically secreted in our experiments. Although there was some suppression of IL-12, levels remained substantially higher than TLR-stimulation alone (Fig. 2*c*) in contrast to the ablation of IL-12 in p38i moDC.

### Opposing role of p38 is mediated through MK2

Using a small molecule inhibitor of MK2 we confirmed the role of this molecule in the opposing p38 effect. Blockade of MK2 reduced IL-12 secretion by moDC [Fig. 2*d*(*i*)] and increased IL-12 from myDC, whilst IL-10 decreased in both DC types [Fig. 2*d*(*ii*)]. Thus, by blocking the p38 pathway at two different levels, we confirmed that it exerts opposing roles in myDC and moDC, and established a role for MK2.

### Differential IL-12 gene transcription upon p38 inhibition

Having established opposing roles for the p38 pathway in moDC and myDC, we determined whether this was transcriptionally regulated. It was previously established that peak transcription of all IL-12 subunits occurs 8–12 hr after TLR stimulation. Furthermore, alterations in IL-12 secretion are typically mediated through changes in peak transcription level rather than transcriptional kinetics, irrespective of TLR agonist.^8^,^18^,^34^,^35^ Our previous study established TOP1 as a stable housekeeping gene in moDC^35^ and this was confirmed in myDC (Supporting Information Fig. 2). There was minimal expression of p35, p40 and p19 by immature DC (Fig. 2*e*). As expected, TLR stimulation induced a marked increase in p19, p35 and p40 mRNA in both DC types. Blockade of p38 in moDC decreased p35 mRNA, with minimal change in p40. In complete contrast, blockade of p38 in myDC resulted in a pronounced increase in both p35 and p40 mRNA. The level of p19 mRNA was only moderately increased in both DC types following p38i.

### Differential effect of p38 on Th1-polarization

**Figure 3:**
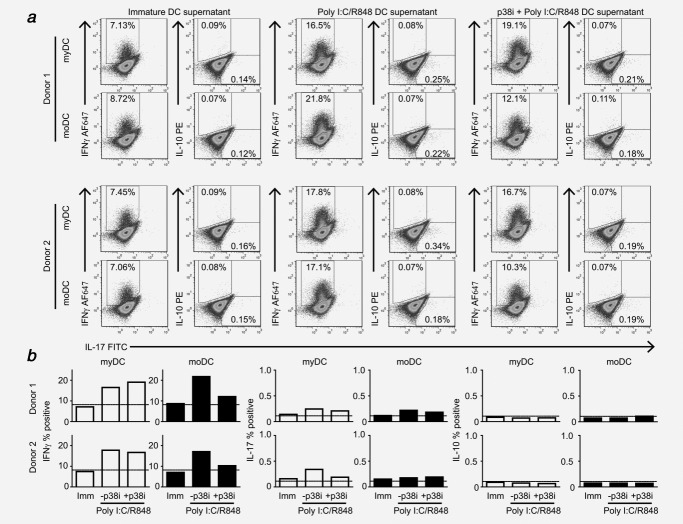
The p38 MAPK plays a differential role in Th1 polarization by myDC and moDC in healthy donors. The effect of differential cytokine production on polarization of naïve Th responses was determined by co-stimulating naïve CD4+CD45RA+ T cells through CD3/CD28 in the presence of 25% supernatant from immature DC (Imm), DC treated with poly I:C/R848 (-p38i) or DC treated with p38 inhibitor SB203580 (10 µM) (+p38i) 1 hr prior to treatment with poly I:C/R848. Rested T cells were re-stimulated with αCD3/αCD28 for 20 h. (*a*) Flow cytometry plots of intracellular cytokine staining for IFNγ, IL-17 and IL-10 for two independent donors. Debris and dead cells were gated out using FS/SS and empty fluorescent channels prior to gating for IFNγ + (AF647), IL-10+ (PE) and IL-17+ (FITC). (*b*) Summary of % positive events for IFNγ, IL-17 and IL-10 for two donors for myDC (open bars) and moDC (filled bars). Dotted line shows the % positive events obtained following αCD3/αCD28 stimulation alone in the presence of blank medium without DC-derived cytokines.

The impact of differential p38 usage on Th-responses was determined by priming naïve CD4+ T-cells in the presence of cytokine-containing supernatant from p38i myDC or moDC. Mature myDC and moDC were equally effective at generating Th1 responses (Fig. 3). In keeping with the loss of IL-12, inhibition of p38 in moDC reduced Th1 responses to baseline levels. In contrast, p38i myDC effectively generated Th1 responses. The effect on Th-17 polarization was more subtle and there was no effect on Treg polarization (IL-10). Importantly, cell-free “mock supernatants,” used to control for carry-over of TLR agonists and MAPK inhibitors, showed no difference to αCD3/αCD28/IL-2 alone (Supporting Information Fig. 3).

### P38 activates Rsk via MK2 in human DC

**Figure 4:**
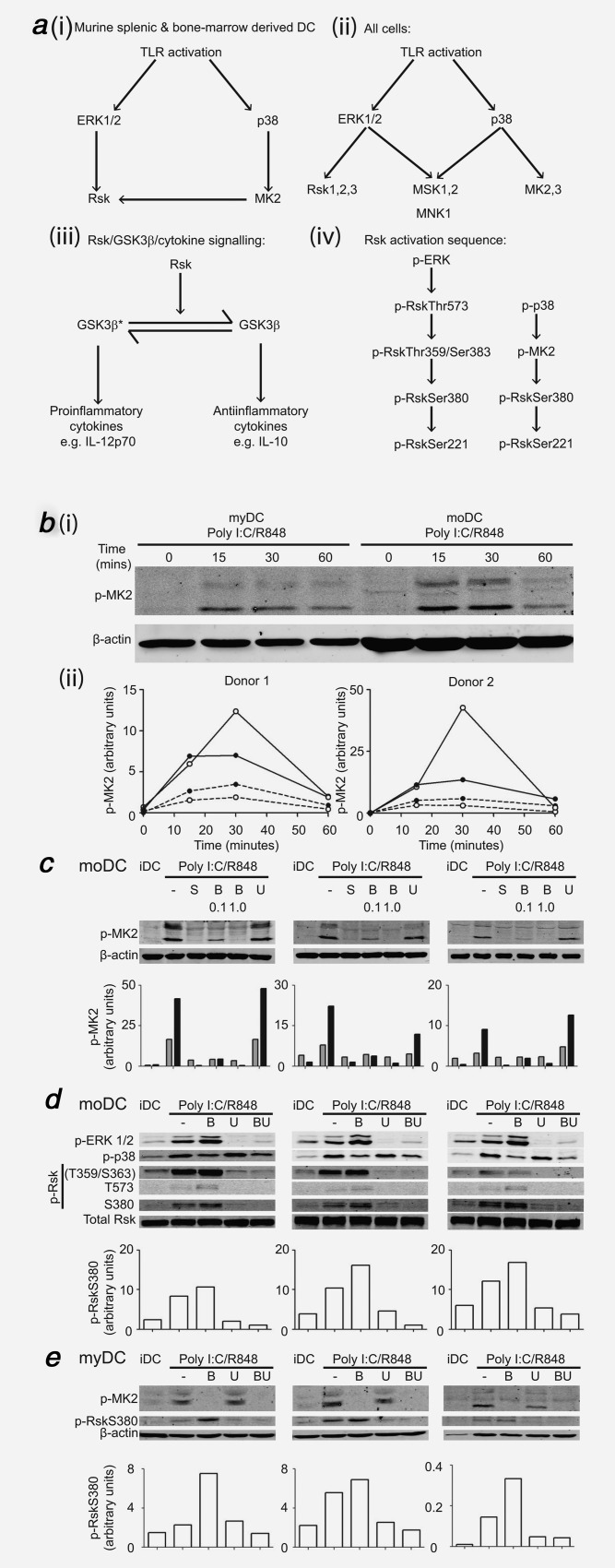
Comparison of intracellular signaling in myDC and moDC after TLR stimulation and effect of blockade of MAPK pathways in healthy donors. (*a*) MAPK/Rsk/GSK3β signaling. (*i*) Activation of Rsk by both ERK and p38 following TLR ligation in murine splenic and bone-marrow derived DC (arrow = activation), (*ii*) Signaling downstream of ERK and p38 in all other cells after TLR ligation where Rsk is only activated by ERK, (*iii*) Rsk drives GSK3β from the active form (GSK3β*) to the inactive form (GSK3β) with downstream effects on IL-12p70/IL-10 secretion, (*iv*) Sequence of phosphorylation/activation of Rsk by phospho-ERK (p-ERK) and phospho-p38 (p-p38). (*b*) Timecourse of MK2 activation following TLR stimulation in myDC and moDC. Donor-matched myDC and moDC (10^5^) were stimulated with poly I:C/R848 for the indicated times before Western blotting. To allow cell-for-cell comparison between myDC and moDC, entire samples were used in a single Western blot and probed for phospho-MK2 (p-MK2) with β-actin as a loading control. Infrared secondary antibodies were used to allow for semi-quantitative analysis normalized to loading control. (*i*) Scanned images for one donor of two. (*ii*) Normalized p-MK2 for two donors, open circles = myDC, filled circles = moDC, dotted line = upper p-MK2 band, solid line = lower p-MK2 band. (*c*) Effect of blockade of p38 or MEK on MK2 activation following TLR stimulation in moDC. MoDC were pre-treated with p38 inhibitors SB203580 10 µM (S), BIRB0796 (B) 0.1 or 1.0 µM or MEK inhibitor UO126 10 µM (U) for 1hr followed by poly I:C/R848 stimulation for 30 min then Western blotted for p-MK2 with β-actin loading control as in (*b*). Shown are scanned images for three independent donors and graphs of normalized p-MK2 (gray bars = upper p-MK2 band, black bars = lower p-MK2 band). (*d*) Effect of blockade of p38, MEK or both on ERK, p38 and Rsk activation in moDC. MoDC were treated and analyzed as in (*c*) for phospho-ERK 1/2 (p-ERK 1/2), phospho-p38 (p-p38), phospho-Rsk (p-Rsk, T359/S363, T573 and S380 residues) with total Rsk as loading control. Pre-treatment was with p38 inhibitor BIRB0796 1.0 µM (B), MEK inhibitor UO126 10 µM (U) or both (BU). Shown are scanned images for three independent donors and graphs of normalised p-RskSer380 for each donor. (*e*) Effect of blockade of p38, MEK or both on activation of MK2 and Rsk at Ser380 in myDC. MyDC (three donors) were treated, harvested and analyzed as in (*c*) for p-MK2 and p-RskS380 with β-actin as loading control. Pre-treatment was with p38 inhibitor BIRB0796 1.0µM (B), MEK inhibitor UO126 10µM (U) or both (BU).

Having established that the differential effect of p38 signaling was mediated *via* MK2, we searched for candidate mechanisms. In murine DC, Watts and coworkers described a unique mechanism of Rsk phosphorylation by p38-MK2, where blockade of both p38 and ERK was required to fully prevent Rsk activation upon TLR stimulation [Fig. 4*a*(*i*)],^36^ in contrast to all other cell types where Rsk is activated exclusively through MEK-ERK [Fig. 4*a*(*ii*)]. This is important as GSK3β, a downstream target of Rsk, is involved in IL-10 and IL-12 regulation [Fig. 4*a*(*iii*)].^37^ To date this p38-MK2-Rsk link has not been demonstrated in human DC and we hypothesized that its absence in human moDC could account for our findings.

In order to study signaling downstream of p38 we first established the time-course of MK2 activation. Immature myDC and moDC did not express phospho-MK2 [Fig. 4*b*(*i*,*ii*)], however MK2 phosphorylation was induced within 15 min of poly I:C/R848 treatment. In moDC, phospho-MK2 levels plateaued after 15 min, and returned to near baseline by 1 hr. In myDC, MK2 phosphorylation peaked at 30 min before decreasing. Although the absolute level of MK2 activation per cell was greater in moDC than myDC [Fig. 4*b*(*i*)], when corrected for cell size using β-actin [Fig. 4*b*(*ii*)], myDC appeared to have more prominent MK2 activation than moDC.

We confirmed that the inhibitors were blocking downstream p38 signaling by measuring MK2 phosphorylation in moDC (Fig. 4*c*). Treatment with SB203580 or BIRB0796 1.0 µM prevented TLR-induced MK2 phosphorylation, whereas 0.1 µM BIRB0796 did not fully inhibit MK2 activation. As expected, MEKi did not affect phospho-MK2 levels (Fig. 4*c*) or p38 activation (Fig. 4*d*) but ablated ERK phosphorylation (Fig. 4*d*).

ERK sequentially phosphorylates Rsk at multiple residues before downstream signaling occurs, whilst p38 leads directly to phosphorylation of the S380 residue in DC, bypassing earlier steps [Fig. 4*a*(*iv*)].^36^ RskS380 phosphorylation is the penultimate step before downstream signaling occurs and is the point at which p38 and ERK activation of Rsk converge in murine DC. Poly I:C/R848 induced phosphorylation of ERK, p38 and Rsk at all residues studied (Fig. 4*d*). Inhibition of p38 increased ERK and Rsk phosphorylation at all residues (presumably due to loss of negative feedback). Rsk phosphorylation at T359/S363 and T573 was prevented by UO126, in keeping with the sequence of phosphorylation events.^36^ Residual phosphorylation of RskS380 remained following UO126 treatment, and this was reproducibly (but subtly) further reduced by BIRB0796 (Fig. 4*d*).

In human myDC, we found that TLR-dependent phosphorylation of MK2 and RskS380 was similar to moDC (Fig. 4*e*). BIRB0796 prevented MK2 phosphorylation but increased phospho-RskS380. The residual level of phospho-RskS380 after MEKi was small, and whilst it reduced further with simultaneous p38i, this effect was subtle. Importantly, there was no difference in the pattern of RskS380 phosphorylation between moDC and myDC. Nevertheless, we demonstrate for the first time that the p38-MK2-Rsk signaling link exists in human DC, though p38 appeared to play a much lesser role in Rsk activation than in murine DC.

### P38 inhibition permits normal IL-12 secretion in myDC from advanced cancer patients

**Figure 5:**
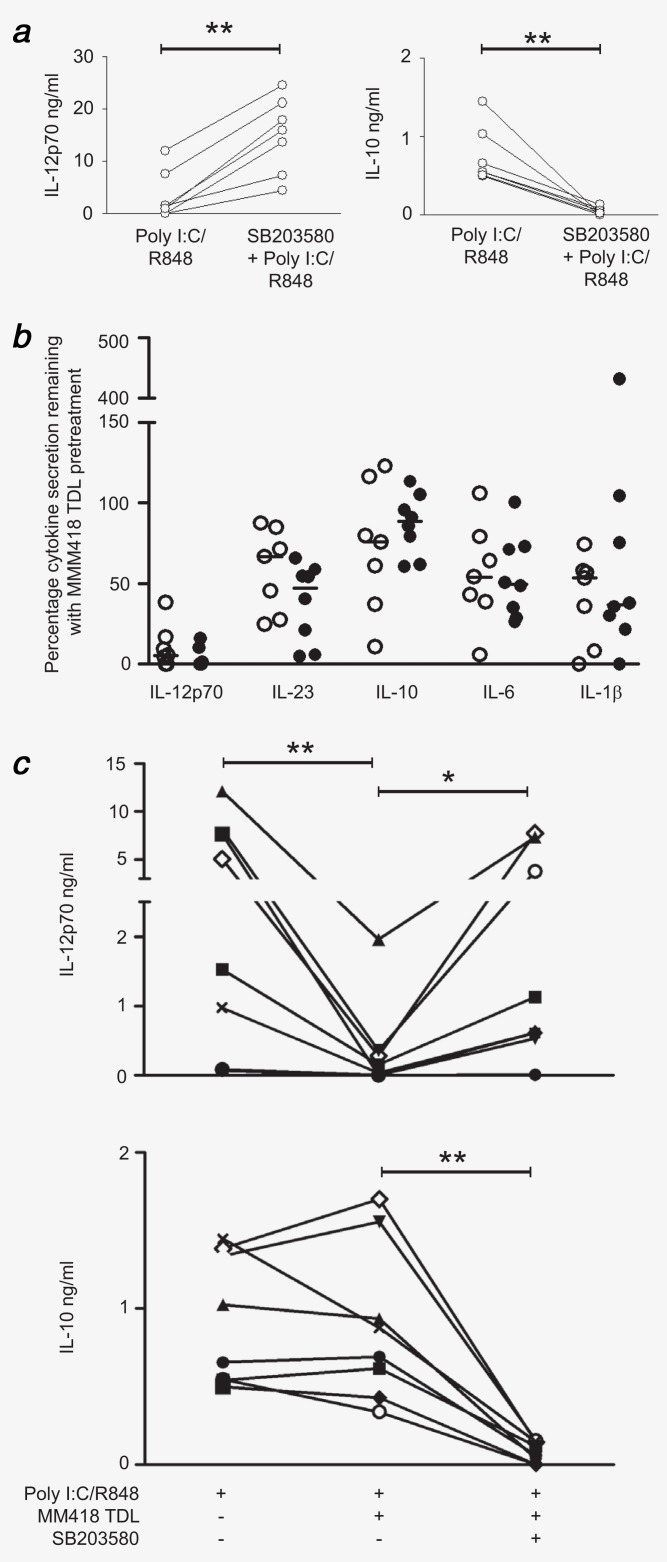
Effect of melanoma lysate with or without p38 inhibitor on cytokine production by myDC from cancer patients. (*a*) MyDC (*n* = 7) from advanced cancer patients were treated in triplicate with p38 inhibitor SB203580 prior to poly I:C/R848. At 24 hr cytokine secretion was analyzed by ELISA. Shown is mean secretion of IL-12p70 and IL-10 for each patient plotted separately with poly I:C/R848 alone and with pre-treatment with MAPK inhibitor. (*b*) MyDC from fully resected (open circles, *n* = 7) and Stage 4 (filled circles, *n* = 8) cancer patients were treated for 1 hr with MM418 TDL before stimulation for 24 hr with poly I:C/R848. Cytokine secretion as a percentage remaining compared to stimulation without pre-treatment with TDL was calculated for each donor. (*c*) MyDC (individual symbols for each patient, *n* = 8) from advanced and fully resected cancer patients were treated as in (*b*) but with 1 hr pre-treatment with p38 inhibitor SB203580 prior to TDL treatment and TLR stimulation. Cytokine production (IL-12p70 and IL-10) for each condition for each donor is plotted. *p* < 0.05 (*) and *p* < 0.005 (**).

Having established a differential role for the p38 pathway in healthy donor DC IL-12 production, we explored whether this still applied in advanced cancer patients. We found that p38i (SB203580) was still able to enhance IL-12 and suppress IL-10 in advanced cancer patients (Fig. 5*a*, *p* = 0.0015 for IL-12, *p* = 0.0025 for IL-10) without affecting other cytokines (not shown). Importantly, in those patients with undetectable baseline secretion, p38i increased IL-12 production to at least normal baseline levels.

### Melanoma lysate suppresses IL-12 in myDC; restoration by p38i

Previously, we showed that exposure of moDC to melanoma TDL impaired IL-12 and Th-1 responses.^18^ Since TDL is used for antigen loading in DC vaccination protocols, we established whether cancer patient myDC were similarly susceptible. In all cancer patient myDC treated with melanoma TDL, suppression of IL-12 was seen (Fig. 5*b*). Levels of other cytokines were less affected, though there was some suppression of IL-23, IL-1β and IL-6. Treatment with p38i (SB203580) prior to TDL pulsing restored the ability of myDC to secrete IL-12 in 6/8 cancer patients and still suppressed IL-10 in all donors (Fig. 5*c*). Thus, enhancement of IL-12 production in myDC due to p38 inhibition is potent enough to overcome the severely suppressive effects of TDL treatment, at the same time as continuing to suppress IL-10 secretion.

## Discussion

We demonstrate that cancer patients have sufficient numbers of myDC to be a viable immunotherapy option. Several studies have enumerated circulating DC with mixed findings, some showing depressed number in advanced cancer and others not,^38^–^42^ leaving this question unresolved. It has previously been difficult to accurately and directly quantify circulating DC but the identification of specific markers and development of reagents allowing direct enumeration make accurate quantification possible. In this study with a limited number of patients, there were sufficient myDC to harvest for cellular therapy, with no difference between resected and Stage 4 groups.

Cytokine secretion was also normal in the majority of patients. To our knowledge, there has been no previous study of the cytokine profile of purified cancer patient myDC, eliminating potential modifying effects of other cell types as would be the case with un-purified cells. We describe for the first time that the p38-MK2 pathway negatively regulates IL-12 in human myDC. This contrasts with the established positive role for p38 in moDC for IL-12. Our findings have implications for the utilization of myDC in cancer immunotherapy and for use of p38i in cancer and autoimmunity. We demonstrated that enhancement of IL-12 after p38i occurs in myDC from advanced cancer patients, including in those with severely impaired cytokine production. This negative role for p38 in myDC was surprising, given its conventionally accepted role in inflammation and the fact that moDC rely on p38 for IL-12 and consequent Th1 polarization.^11^–^14^ More recently, however, it has been shown that p38 signaling can lead to anti-inflammatory responses^43^ and that the requirement for p38 during induction of inflammation is tissue- and stimulus-specific.^44^ We obtained similar results with two selective p38 inhibitors, SB203580 and BIRB0796, and an MK2 inhibitor further confirmed the p38-dependence of this observation.

The differential involvement of the link allowing p38 to activate Rsk (mediated through MK2)^36^ could have accounted for the opposing roles of p38 in myDC and moDC. However, the pattern of Rsk phosphorylation with p38i, MEKi or dual blockade was similar in both DC types, discounting this as the mechanism. It would appear that the p38-Rsk signaling link is less important in human DC than murine DC, as only a small amount of Rsk activity remained when MEK-ERK was blocked and further reduction with both inhibitors was small. MK2 has multiple downstream ligands, including HSP27, LSP1, CREB, ATF1, SRF, tyrosine hydrolase and TTP^45^ and is involved in a variety of cellular processes. Other than the p38-MK2-Rsk-GSK3β candidate mechanism eliminated in this study, a review of the literature does not reveal any alternate mechanisms to explore. Further work to establish the down-stream targets of MK2 responsible for the opposing effects on IL-12 production between myDC and moDC will require detailed analysis of transcription factor binding to the differentially affected IL12A gene.

Whilst the decrease in IL-12 from moDC after p38i suppressed Th1 polarization, the increase in IL-12 in myDC did not further induce Th1 responses in healthy donors. It could be that levels of IL-12 were already optimal, or IL-10 may play a role. IL-10 is secreted earlier than IL-12, and the experimental procedure requires removal of TLR agonists/drugs to prevent their direct impact on T-cells. As the time frame for washing cells overlapped with secretion of IL-10 any beneficial effect of suppressing IL-10 may not be evident in the T-cell stimulations. In patients with severely impaired IL-12 production, p38i restored secretion to normal and it is possible that p38i could recover deficient Th1 polarization in these patients. Due to restrictions on venepuncture (hence leucocytes) in our advanced cancer patients, it was not possible to undertake Th1 polarization studies. However, this will form an important part of our pre-clinical study and the activation of T-cell responses by DC:T-cell co-cultures will be determined.

Treatment of cancer patient myDC with TDL suppressed IL-12. Firstly, this showed that the Stage 4 patients in this study were not “resistant” to the suppressive effects of tumor, but rather that their cells had not been significantly impaired *in vivo* since their baseline cytokine secretion was normal. Secondly, it confirmed our previous observations regarding the detrimental effect of freeze-thaw TDL as an antigen source.^18^ TDL is attractive since it provides multiple antigens and is not restricted to specific HLA types. Recent work has focused on generating more immunogenic lysates using heatshock^46^ or radiation^47^ prior to lysis or the conjugation of virus-like particles to lysate before DC loading,^48^ but our study confirmed that conventional freeze-thaw TDL is suppressive and thus should be treated with caution as an antigen source.

Our study has important implications for the efficacy of p38i currently being tested in autoimmune/inflammatory conditions. If p38i *in vivo* mirrors the observed *ex vivo* effects then systemic administration of p38i may increase IL-12 production, contrary to the intended anti-inflammatory effect. That MK2 blockade produced a similar cytokine profile in myDC as p38i raises the possibility of targeting MK2 to support Th1 responses. Clinical use of p38i has been hampered by side effects of liver toxicity and rashes, which may be due to off-target effects on other pathways, or to blockade of all downstream functions of p38. In order to alter myDC IL-12 production and hence enhance Th1 polarization it may be sufficient to block only MK2, avoiding effects on other facets of p38 signaling. Further work is required to explore in more detail the effects of MK2i on myDC. In cancer, p38i are being tested for their direct anti-tumor effect^19^ and our observations raise the possibility of combining systemic p38i with targeted immunotherapeutic approaches.

Using whole peripheral blood as a source may not yield adequate cell numbers as 500 ml would typically yield 1.5 × 10^6^ myDC. It has been shown that CD1c+ myDC can be isolated in a GMP-approved manner for clinical use in sufficient numbers from a leucopheresis procedure, with a mean of 13.8 × 10^9^ PBMC and 39.5 × 10^6^ CD1c+ DC obtained with mean 86% purity.^49^ The number of myDC which could be administered per vaccination may be lower than in moDC clinical trials (which typically administer 5 × 10^6^ to 5 × 10^7^ moDC per vaccination); however if the pre-clinical data on myDC regarding increased ability to migrate^5^–^7^ and enhanced T-cell stimulatory capacity^4^ is confirmed, the net “immune potency” per vaccination may be substantially improved despite smaller cell numbers. We are therefore embarking on a pre-clinical feasibility study to establish the necessary systems for isolation of sufficient myDC from cancer patients, with the aim of initiating a phase I study of adoptively transferred myDC treated with p38i.

In cancer patients, circulating myDC are present in sufficient numbers to allow their use as a therapeutic product. As a group their cytokine secretion after TLR stimulation is normal and even in individuals with impaired cytokine production, p38i restores IL-12 to normal levels. Further development of p38-inhibited circulating myDC as a therapeutic strategy for cancer is warranted.
