# Supplementary material for: Novel function for the p38-MK2 signaling pathway in circulating CD1c+ (BDCA-1+) myeloid dendritic cells from healthy donors and advanced cancer patients; inhibition of p38 enhances IL-12 whilst suppressing IL-10
Source: Int J Cancer. 2013 Nov 20;134(3):575–86. doi: 10.1002/ijc.28398 (PMC4298783; doi:10.1002/ijc.28398)
Supplement: Supplementary file 1 — Supplementary Information [file ijc0134-0575-sd1.doc]

Supplementary figure 1

**Gating and analysis strategy for DC enumeration.**

Two samples of 300µl of whole blood were stained with anti-CD19 PECy5 and anti-CD14 PECy5 and either IgG1FITC and IgG2aPE or anti-CD303 FITC and anti-CD1c PE for 15 minutes prior to lysis of red cells, washing and resuspension in 500µl of FACS buffer. Countbeads were added immediately prior to acquisition. Compensation was established using Compbeads stained for FITC, PE and PECy5. **(A)** For analysis of specifically labelled sample (bottom row), debris was gated out on FS/SS plot (ii, gate P2) and the remaining events plotted on a SS/PECy5 graph (iii). A further gate was applied to exclude CD14 and CD19 high events and SS high events (P3). This gate was drawn on the last complete log contour for each of the excluded populations in order to be objective regarding placement. Remaining events were plotted on a FITC vs PE graph (iv) and a box-gate used to count myDC (CD1c+, P4) and pDC (CD303+, P5) events. The same gating strategy was used on the control sample (top row) in order to correct for nonspecific binding. Once the number of myDC and pDC events captured was defined for each sample, the absolute number/ml of whole blood was calculated. On an FL3/FS plot (i) a box-gate was used to determine the number of count beads acquired (P1) and the volume of the initial sample acquired was calculated using the following formula:

Volume of blood acquired (ml) = (number of count beads acquired/number of count beads added) *0.3

For each sample, the total number of myDC or pDC events was used to calculate the number per ml of blood using the following formula:

Number of cells/ml blood= number acquired/volume of blood acquired

The final number of DC per ml of blood was then calculated by subtracting the number of myDC or pDC events per ml of blood in the control sample from the number in the sample stained with specific antibodies.

Validation of staining and gating strategy:

**(B)** Triplicate staining of a single sample from each of 3 donors, analysis by a single operator. Shown is calculated cell count for each staining for each donor, line is mean and indicated is coefficient of variation (cv) for myDC and pDC. **(C)** Triplicate analysis (three operators) of singlicate staining for 3 donors, shown is 1 experiment of 2 with calculated cell count by each operator for each donor, line is mean and indicated is coefficient of variation (cv) for myDC and pDC.

Supplementary Figure 2

**Robustness of TOP1 as a housekeeping gene in myDC and moDC.**

Little published data exists concerning reliability of housekeeping genes in myDC. Therefore we examined the utility of the TOP1 gene, previously determined by us to be a useful gene for Q-PCR studies in moDC[^35^](#_ENREF_35). mRNA was extracted from resting DC, DC stimulated with poly I:C/R848, or DC pre-treated with SB203580 prior to poly I:C/R848. Mean cycle threshold (CT) values +/- standard deviation for TOP1 expression in myDC (open bars) and moDC (filled bars) from triplicate determinations of 3 independent donors are shown.

Supplementary Figure 3

**Control experiments showing no adverse effect of carry-over of MAPK inhibitors to T cell cultures.** Cell-free ‘mock’ wells were established and treated identically to those containing DC by the addition of MAPK inhibitors and TLR agonists. Wells were washed simultaneously with cell-containing wells. Naïve CD4+CD45RA+ T-cells were stimulated with antibodies to CD3 and CD28 in the present of 25% ‘mock’ supernatants. After resting and re-stimulation for 20h the expression of cytokines was determined by intracellular staining and flow-cytometry. Conditions were αCD3/CD28 alone (-) or with addition of supernatant from mock immature DC (‘iDC’, GM-CSF only), mock mature DC (‘mDC’, GM-CSF + poly I:C/R848) or mock mature DC + SB203580 pre-treatment for 1hr (‘mDC +p38i’, GM-CSF + SB203580 + poly I:C/R848).
